# Clinical Outcomes of Volumetric Modulated Arc Therapy Following Intracavitary/Interstitial Brachytherapy in Cervical Cancer: A Single Institution Retrospective Experience

**DOI:** 10.3389/fonc.2019.00760

**Published:** 2019-08-16

**Authors:** Yanzhu Lin, Yi Ouyang, Kai Chen, Zhiyuan Lu, Yonghong Liu, Xinping Cao

**Affiliations:** ^1^State Key Laboratory of Oncology in South China, Department of Radiation Oncology, Sun Yat-sen University Cancer Center, Collaborative Innovation Center for Cancer Medicine, Guangzhou, China; ^2^Department of Oral and Maxillofacial Surgery, Stomatology Medical Center, Guangzhou Women and Children's Medical Center, Guangzhou, China

**Keywords:** cervical cancer, volumetric modulated arc therapy, brachytherapy, toxicity, radiotherapy

## Abstract

**Purpose:** To evaluate treatment outcomes and toxicity in patients with cervical cancer (CC) treated with volumetric modulated arc therapy (VMAT), followed by three-dimensional high-dose-rate intracavity combined with interstitial brachytherapy (IC/IS BT) compared with intensity-modulated radiation therapy (IMRT) treatment.

**Materials and Methods:** A total of 398 patients with stage IA–IVB CC treated with definitive radiotherapy with or without chemotherapy were retrospectively analyzed (331 VMAT and 67 IMRT). A total prescription dose of 45–50 Gy was delivered to pelvic field with VMAT/IMRT in 25/28 fractions, with five fractions per week. Every patient further received IC/IS BT for four to six 6.0-Gy fractions. Local control (LC), disease-free survival (DFS), overall survival (OS), and distant metastasis-free survival (DMFS) rates were calculated. Acute hematotoxicity and late toxicity were recorded.

**Results:** The median follow-up period was 25.47 (range, 0.93–58.93) months for the VMAT and 35.07 (4.8–90.37) months for IMRT. The 3-year OS, DFS, LC, and DMFS rate were 80.5, 65.4, 88.7, and 78.1% in VMAT group, and 76.2, 76.4, 83.1, and 86.1% in the IMRT group, respectively. No significant differences were found between VMAT and IMRT groups for OS, DFS, LC, and DMFS rate. However, patients in the VMAT group had lower incidence of chronic enterocolitis complication (26.6 vs. 38.8%, *p* = 0.004). In addition, a total of 3 (0.9%) patients developed grade 3 chronic cystitis, and 7 (2.1%) patients developed grade 3 or greater chronic enterocolitis in VMAT group.

**Conclusion:** VMAT combined with IC/IS BT can result in satisfactory curative outcomes and low incidences of late radiation enterocolitis and cystitis in CC treatment.

## Introduction

Cervical cancer (CC) is the forth most common cancer in women worldwide (1), with an incidence of 98,900 cases and 30,500 deaths annually in China (2). Radiotherapy (RT) has been used for more than 100 years to treat CC. Definitive RT, including external beam RT (EBRT) and brachytherapy (BT) combined with concurrent chemotherapy, is the main treatment modality for patients with stage IIB–IVA CC (3).

Intensity-modulated RT (IMRT) is a technique that enables greater conformity and uniform dose distribution to a tumor, thus reducing damage to surrounding normal tissues (4). IMRT can be used to achieve excellent treatment outcomes for CC as well as to effectively reduce side effects such as radioactive cystitis and enteritis (5). Volumetric modulated arc therapy (VMAT) is an advanced form of IMRT; VMAT results in greater conformity and shorter treatment delivery time compared with IMRT (6). At our institution, definitive VMAT has been used since 2012 for treating patients with CC. However, data on the efficacy of definitive VMAT in the treatment of CC are limited.

BT is an important form of definitive RT used for treating CC; the modality has evolved from a two-dimensional (2D) planning to a three-dimensional (3D) image-guided BT (IGBT) technique (7). IGBT can be administered using an intracavitary (IC), interstitial (IS), or combined IC/IS approach (8). At our institution, 3D computed tomography (CT) image-based IC/IS for the definitive treatment of CC after EBRT has been used since 2013. However, data on the efficacy of 3D image-guided IS/IC BT for the definitive treatment of CC remains unclear.

To provide further data, we conducted a retrospective study to verify the pattern of administering combined IC/IS BT with VMAT for the definitive treatment of CC, compared with IMRT at our institution. The present study reviewed the efficacy and toxicity of VMAT and IMRT for CC patients during our early experience.

## Methods and Materials

### Patient Cohort

We retrospectively reviewed 398 women who were diagnosed with stage IA–IVB CC and treated with definitive radiation at the Sun Yat-sen University Cancer Center. There were 67 patients with CC treated with definitive IMRT between December 2010 and September 2012. Thereafter, 331 patients were treated VMAT form May 2013 to May 2017. We included patients who (1) had histologically confirmed CC; (2) were newly diagnosed with CC and had not received any treatment before; (3) were staged according to a standard protocol that included gynecological examination, routine blood examination, squamous cell carcinoma (SCC) antigen test, pelvic magnetic resonance imaging (MRI), chest and abdomen CT, and positron emission tomography (PET)/CT staging (performed in some patients); (4) were treated with curative intent with a combination of definitive radiation (with or without concomitant chemotherapy) followed by HDR BT; and (5) had a Karnofsky performance status > 70. We reviewed patients' clinical records and obtained the following information: age at diagnosis, International Federation of Obstetricians and Gynecologists (FIGO) stage, histological type, tumor size on MRI, pre-treatment SCC, last date of patient follow-up, date of recurrence, and time of death. This study was approved by the Ethical Committee of the Sun Yat-sen University Cancer Center (protocol number B2019-074-01). Written informed consent was obtained from the participants of this study.

### Radiotherapy

#### CT Simulation

Before simulation, patients were instructed to empty the rectum. All patients were immobilized in a supine position within a thermoplastic shell, with their pelvic cavity maintained in a neutral position. Intravenous contrast-enhanced CT simulation was performed at 3-mm slice thickness on the whole abdomen and pelvic region by using a CT simulator.

#### Target Volumes

Reconstructed CT images were transmitted to a Monaco treatment planning system (TPS). The gross target volume included all grossly enlarged lymph nodes with a short diameter of ≥1 cm and regional metastatic lymph nodes on imaging findings or as determined by PET/CT findings. The clinical target volume (CTV) included the cervix, whole uterus, parametrium, upper part of the vagina, and pelvic lymphatic drainage area (common, internal, and external iliac; obturator; and presacral). Inguinal lymph nodes were included if lower one-third vaginal involvement was observed. In patients with common iliac metastatic lymph nodes, para-aortic irradiation was administered. The planning target volume (PTV) was generated using a 7-mm margin relative to the CTV. Concurrent chemotherapy (a weekly cisplatin dose of 40 mg/m^2^) was systematically administered except in case of contraindication, elderly age (>70 years), severe comorbidity, performance Status score >2 or refusal.

#### Treatment Planning and Delivery

Patients were administered 1.8–2 Gy per daily fraction five times a week. The prescribed dose of 45–50 Gy was delivered to the PTV with whole-pelvis VMAT or IMRT in 25–28 fractions, and a lymph node boost up to 60–70 Gy was delivered to the gross residual mass in case of lymph node metastasis. Target planning constraints were V_95_ ≥ 95% and V_110_ < 20%. Organs at risk (OARs) to be contoured were the bladder, small intestine, rectum, kidney, spinal cord, and femoral head. Dose constraints for the OARs were as follows: bladder V_45_ < 35%, rectum V_45_ < 60%, kidneys V_18_ < 20%, femoral head V_30_ < 15%, bowel V_40_ < 30%, and spinal cord D1cc ≤ 40 Gy.

### Image-Guided BT

High-dose-rate BT afterloading technique (Flextron) with iridium-192 was given after the completion of RT. Before May 2013, 67 patients in IMRT group were treated with 2D BT, thereafter, 331 patients underwent 3D CT image-guided BT. The size and location of a residual tumor were preliminarily assessed based on the findings of pelvic MRI and gynecological examination; then, the location for placing an intrauterine catheter, as well as the number and position of interstitial needles, were determined. A Foley catheter was inserted, and the bladder was filled with 300 cm^3^ of sterile saline. First, an intrauterine catheter was inserted into the uterine cavity; then, needles were inserted by hand and fixed by inserting iodoform gauze. CT scans were conducted after the first insertion to adjust the depth and position of the inserted needles and identify uterine perforation. After completion of catheter and needle implantation, 3-mm-thick CT scans were obtained again until the optimal positioning of the inserted needles was determined. Images were then transferred to a 3D TPS (Elekta). Contour delineation of the high-risk CTV and OARs (bladder, rectum, and sigmoid) was performed. A median prescribed dose of 30 Gy was delivered once a week (range: 24–36 Gy; 4–6 fractions). For IMRT group, BT was delivered to point A at a dose of 6 Gy in 4–6 fractions weekly.

### Toxicity

Acute toxicity was assessed weekly during the course of radiotherapy and 1 month after radiotherapy. Late toxicity was defined as adverse events occurring after 90 days from completion of BT. Acute and late toxicity was graded based on the site and severity observed during follow-up according to the Radiation Therapy Oncology Group criteria and European Organization for Research and Treatment of Cancer (9).

### Follow-Up and Statistical Analysis

Patients were followed up at 3-month intervals in the first year and at 6-month intervals in the following years. The final follow-up was on October 31, 2018. All outcomes were measured from the date of end of BT to the time of the given event. Local relapses were defined as any recurrence in the vagina, cervix, uterus, or parametria. Pelvic recurrence was defined as any local or nodal pelvic recurrence. Regional relapses were defined as any recurrence in the pelvis or in the para aortic lymph node area. Statistical analysis was performed using SPSS (version 22, SPSS Inc, Chicago, IL). The Kaplan–Meier method and log-rank test were employed to estimate overall survival (OS), disease-free survival (DFS), distant metastasis-free survival (DMFS), and locoregional control (LC) rates. Clinical toxicities were compared using a χ^2^ test between two groups. Differences between groups were examined using the log-rank test. Statistical significance was defined as *P* < 0.05.

## Results

### Patients' Characteristics

Table 1 lists the detailed characteristics of patients. Patients' median age at diagnosis was 57 (range, 27–84) years for VMAT group and 59 (range, 29–85) years for IMRT group. Most of the patients in VMAT (*n* = 312, 94.3%) had squamous cell carcinoma. The FIGO stage distribution in VMAT was as follows: IA (*n* = 1, 0.3%), IB (*n* = 14, 4.2%), IIA (*n* = 41, 12.4%), IIB (*n* = 138, 41.7%), IIIA (*n* = 29, 8.8%), IIIB (*n* = 91, 27.5%), IVA (*n* = 2, 0.6%), and IVB (*n* = 15, 4.5%). Concurrent chemotherapy was administered to 230 (69.5%) patients in VMAT group, including 197 patients with stage IIB-IVB. The median primary cervical tumor width at diagnosis identified using MRI was 47 (range, 10–100) mm in VMAT group, and 48 (range, 10–93) mm in IMRT group. The MRI width was <40 nm and ≥40 nm in 126 (38%) and 205 (62%) patients in VMAT group, respectively. The median pre-treatment SCC-Ag level was 7.1 (range, 0.1–70) ng/mL in VMAT group and 5.4 (range, 0.8–70) ng/mL in IMRT group.

**Table 1:** Patients and tumor characteristic.

**Characteristics**	**VMRT group (*n* = 331)**	**IMRT group (*n* = 67)**
Median age (years)	57 (27–84)	59 (29–85)
<60	190 (57.4%)	40 (59.7%)
≥60	141 (42.6%)	27 (40.3)
**FIGO stage**		
IA	1 (0.3%)	0 (0%)
IB	14 (4.2%)	6 (8.9%)
IIA	41 (12.4%)	9 (13.4%)
IIB	138 (41.7%)	26 (38.9%)
IIIA	29 (8.8%)	6 (8.9%)
IIIB	91 (27.5%)	20 (29.9%)
IVA	2 (0.6%)	0 (0%)
IVB	15 (4.5%)	0 (0%)
**Histology**		
Squamous cell	312 (94.3%)	63 (94.4%)
Adenocarcinoma	8 (2.4%)	1 (1.4%)
Adenosquamous	5 (1.5%)	1 (1.4%)
carcinoma		
Others	6 (1.8%)	2 (2.8%)
**Chemotherapy**		
Yes	230 (69.5%)	51 (76.1%)
No	101 (30.5%)	16 (23.9%)
**Pre-treatment MRI-based maximal diameter (mm)**		
Median (range)	47 (10–100)	48 (10–93)
<40 mm	126 (38%)	15 (22.4%)
≥40 mm	205 (62%)	52 (77.6%)
**Pre-treatment SCC level (ng/ml)**		
Median (range)	7.1 (0.1–70)	5.4 (0.8–70)

*FIGO, International Federation of Gynecologic Oncology; SCC, squamous-cell carcinoma antigen*.

### Clinical Outcomes

The median follow-up period in VMAT group was 25.47 (range, 0.93–58.93) months. For VMAT group, at the time of the analysis, 276 patients were alive and 55 patients died during follow-up. Among the patients who were alive at the final follow-up in VMAT group, 225 had no evidence of disease and 51 experienced a relapse with or without active treatment. The median time interval from the end of treatment to tumor relapse was 6.5 (range, 0.43–38.47) months in VMAT group. A total of 90 (27.2%) patients developed tumor recurrence in VMAT group. Among them, 84 patients (93.3%) experienced tumor relapse in the first 2 years, 35 (38.9%) patients had local recurrence only, 44 (48.9%) patients had pelvic relapses, 50 (55.6%) patients had regional relapses, 62 (68.9%) patients had distant metastases (Table 2).

**Table 2:** Distribution of tumor recurrences for patients with cervical cancer in VMAT group.

**Recurrences**	**Number of patients (%)**
Overall	90 (27.2)
Local	35 (38.9)
Pelvic	44 (48.9)
Regional	50 (55.6)
Distant	62 (68.9)

**Figure 1:**
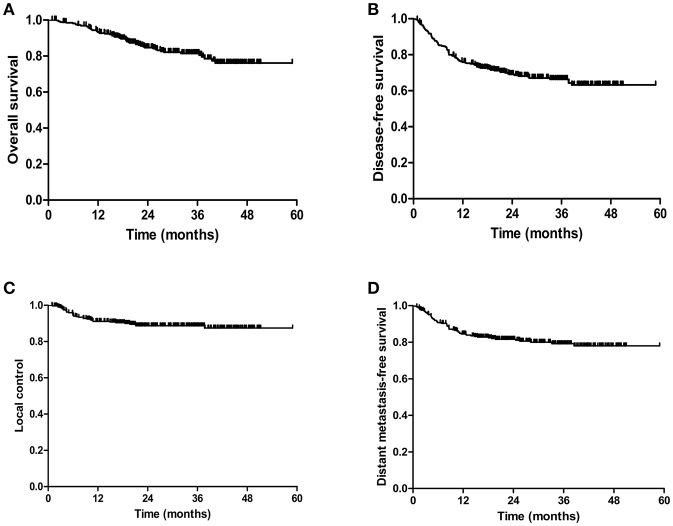
Kaplan-Meier estimated of the overall survival (OS) **(A)**, disease-free survival (DFS) **(B)**, local control (LC) rates **(C)**, and distant metastasis free survival (DMFS) **(D)** of cervical cancer patients treated with VMAT.

**Figure 2:**
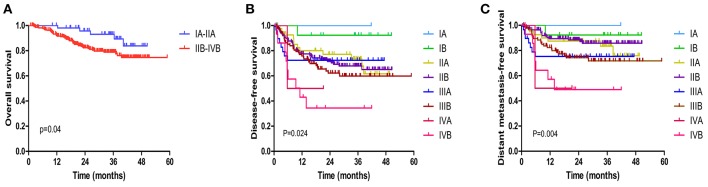
Kaplan-Meier estimated of the overall survival (OS) **(A)**, disease-free survival (DFS) **(B)**, and distant metastasis free survival (DMFS) **(C)** according to the FIGO stage treated with VMAT.

As illustrated in Figure 1, the 3-year OS rate of VMAT group was 80.5%. The 3-year DFS rate was 65.4%. The 3-year LC rate was 88.7%. The 3-year DMFS rate was 78.1%. Clinical outcomes according to FIGO stage are presented in Table 3. The crude local, pelvic and regional control rates were 89.4, 86.7, and 84.9% in VMAT group, respectively. LC at 3 years was 100% in IA, 100% in IB, 92% in IIA, 86.8% in IIB, 96.2% in IIIA, 87.7% in IIIB and 78.5% in IVB stage in VMAT group (Table 3). The 3-year OS rates for patients in VMAT group with stage IA, IB, IIA, IIB, IIIA, IIIB and IVB disease were 100, 100, 85.7, 79.2, 75.6, 82, and 60.1%, respectively (Table 3). Patients in VMAT group with Stage IA-IIA cervical cancer had significantly better OS (hazard ratio [HR], 0.51; 95% CI, 0.26–0.98, *p* = 0.04) rates compared with Stage IIB-IVB cervical cancer patients (Figure 2A). The results of the univariate analysis revealed there was a significant difference in DFS and DMFS among women of different FIGO ages (*p* = 0.024, *p* = 0.004, Figures 2B,C).

**Table 3:** Clinical outcomes at 3 years according to stages in VAMT group patients.

**FIGO Stage**	**Number of patients**	**Crude LC rate**	**Crude PC rate**	**Crude RC rate**	**Crude DMFS**	**OS at 3 year**	**DFS at 3 year**	**LC at 3 year**	**DMFS at 3 year**
IA	1	100%	100%	100%	100%	100%	100%	100%	100%
IB	14	100%	100%	100%	92.9%	100%	92.3%	100%	92.3%
IIA	41	90.2%	82.9%	80.5%	82.9%	85.7%	73.5%	92.0%	83.5%
IIB	138	87.7%	86.2%	84.1%	87.7%	79.2%	67.9%	86.8%	85.8%
IIIA	29	96.6%	93.1%	93.1%	75.9%	75.6%	72.4%	96.2%	75.3%
IIIB	91	89%	85.7%	83.5%	75.8%	82%	59.9%	87.7%	71.8%
IVA	2	100%	100%	100%	50%	–	–	–	–
IVB	15	80%	80%	80%	53.3	60.1%	34.5%	78.3%	49.0%
overall	331	89.4%	86.7%	84.9%	81.3	–	–	–	–

The median follow-up period in IMRT group was 35.07 (range, 4.80–90.37) months. At the last follow-up visit, 14 patients died, 13 patients had local recurrence and 12 patients had distant metastasis. The 3-year OS, DFS, LC, and DMFS were 76.2, 76.4, 83.1, and 86.1% in the IMRT group, respectively (Supplemental Figures 1A–D). A comparison of OS, DFS, LC, and DMFS showed no significant differences between VMAT and IMRT (Supplemental Figures 1A–D).

**Figure 3:**
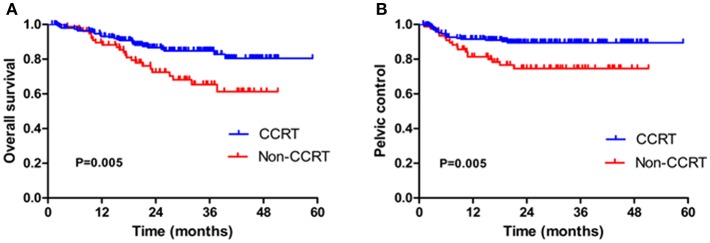
Kaplan-Meier estimated of the overall survival (OS) **(A)** and pelvic control (PC) **(B)** in locally advanced cervical patients (stage IIB-IVB) in VMAT group with concurrent chemoradiotherapy (CCRT) or without CCRT (Non-CCRT).

Concurrent chemoradiotherapy (CCRT) for patients with locally advanced cervical cancer (stage IIB-IVB, *n* = 197) in VMAT group had a significant impact on OS: 3-year OS in the CCRT group was 84.8% compared to 65.4% in the non-CCRT group (HR, 2.403, 95% CI 1.298–4.449, *p* = 0.005, Figure 3A). In addition, CCRT was associated with better PC rate than that of the non-CCRT group for patients treated with VMAT (*p* = 0.005, Figure 3B).

### Toxicity Evaluation

The most frequently observed acute hematologic toxicity, including leukocytopenia, anemia, and erythropenia (Table 4). Overall, patients in VMAT group had a lower incidence of acute anemia than did those in IMRT group (33.5 vs. 65.7%, *p* < 0.001). In VMAT group, the incidence of grade 3 and 4 acute anemia/erythropenia were 3.6 and 0.9%. The percentages of patients in VMRT group who experienced acute grade 3 and 4 leukopenia were 8.5 and 0.6%. Acute leukopenia was common with no differences in both groups (*p* = 0.109).

**Table 4:** Crude incidence of acute hematotoxicity in patients with cervical cancer.

**Grade**	**VMRT group (*n* = 331)**	**IMRT group (*n* = 67)**	***P*-value**
Hgb/Hct			<0.001
0	220 (66.5%)	23 (34.3%)	
1	64 (19.3%)	35 (52.3%)	
2	32 (9.7%)	7 (10.4%)	
3	12 (3.6%)	2 (3.0%)	
4	3 (0.9%)	0 (0%)	
Leukopenia			0.109
0	175 (52.9%)	31 (46.3%)	
1	108 (32.6%)	29 (43.2%)	
2	18 (5.4%)	6 (9.0%)	
3	28 (8.5%)	1 (1.5%)	
4	2 (0.6%)	0 (0%)	

*Hct, hematocrit; Hgb, hemoglobin*.

We examined the presence of late toxicity symptoms. Table 5 summarizes the number of patients with and grades of late toxicity for the entire cohort. At the time of follow-up, the percentages of patients in VMAT group with grade 1 chronic cystitis and enterocolitis were 3.0 and 20.6%, respectively. Furthermore, 20 (6%) patients had grade 2 or higher chronic enterocolitis, whereas 7 (2.1%) patients had grade 2 or higher chronic cystitis in VMAT group. Only three patients experienced grade 4 enterocolitis and no grade 4 chronic cystitis was observed in VMAT group. Moreover, the chronic enterocolitis was significantly lower in the VMAT group (*p* = 0.004).

**Table 5:** Chronic toxicities in patients with cervical cancer.

**Grade**	**VMRT group (*n* = 331)**	**IMRT group (*n* = 67)**	***P*-value**
Cystitis			0.230
0	314 (94.9%)	60 (89.5%)	
1	10 (3.0%)	3 (4.5%)	
2	4 (1.2%)	3 (4.5%)	
3	3 (0.9%)	1 (1.5%)	
4	0 (0%)	0 (0%)	
Enterocolitis			0.004
0	243 (73.4%)	41 (61.2%)	
1	68 (20.6%)	13 (19.4%)	
2	13 (3.9%)	6 (9.0%)	
3	4 (1.2%)	4 (5.9%)	
4	3 (0.9%)	3 (4.5%)	

## Discussion

The use of VMAT for treating CC has increased significantly. However, data on the long-term outcomes and toxicity of definitive VMAT for CC remain insufficient. Herein, we conducted a retrospective institutional experience of treating patients with CC with definitive VMAT verse IMRT. To the best of our knowledge, this is the first study that included a large cohort of patients with CC who were treated with VMAT and CT-based IG/IS BT. Our results revealed that there were no significant differences in OS, DFS, LC, and DMFS between VMAT and IMRT, but VMAT were associated with lower incidence of acute anemia and chronic enterocolitis.

Currently, pelvic EBRT with intracavity BT is the recommended standard treatment for the definitive treatment of CC (10). Although pelvic conventional RT for treating CC results in satisfactory therapeutic outcomes, it is associated with serious acute and chronic toxicity, including small bowel obstruction, enteritis, rectitis, and radiation cystitis (11). Several retrospective studies have shown that the incidence of radiation complications reached 20–30% after EBRT, with the incidences of radiation proctitis and radiocystitis being 10–20% and 3–5%, respectively (10, 12). In addition, because the dose administered in the tumor area cannot be increased in conventional irradiation, the local control rate is low. With the development of computer technology and modern radiation techniques, precision RT has been widely used in clinical practice. IMRT provides satisfactory conformity, which can improve the accuracy of RT to the maximum extent, as well as reduce the occurrence of complications (13). For the treatment of gynecologic cancers, most studies have shown that IMRT improved the conformity of the target area as well as reduced the exposure dose and volume of OARs compared with 3D conformal radiation therapy (3D-CRT) (14, 15). VMAT is a more favorable choice for external radiation therapy in CC because it enables reduced treatment time, thus improving clinical outcomes.

BT is an essential curative element of definitive RT for CC; it enables accurate delivery of a high dose to a tumor while sparing surrounding normal tissues and yields favorable treatment outcomes (16). However, BT is associated with late complications arising in organs adjacent to the cervix, such as the rectum and bladder. Recently, 3D IGBT techniques have been developed for treating gynecologic malignancies that have been shown to exhibit more favorable dosimetry and deliver a decreased dose to nearby OARs, thereby leading to lower toxicity and improved local control (17, 18). Kang et al. (19) retrospectively analyzed and compared the outcomes of 97 patients with CC treated with 3D CT-based high-dose-rate intracavitary RT with those of 133 patients treated with 2D-BT. They reported that the incidence of severe late rectal bleeding decreased from 13 to 2% and LC was significantly improved. Because MRI is the gold standard for CC, MRI-based planning was implemented in BT for CC treatment owing to better delineation of target volumes and identification of OARs. However, a study reported that treatment of locally advanced CC with MR-guided interstitial BT following EBRT was associated with equivalent local control, toxicity, and improved OS, as per the results of the Kaplan–Meier analysis, when compared with CT-guided interstitial BT (20).

Numerous studies have utilized a combined approach of EBRT, followed by BT, as a definitive RT modality for medically inoperable CC and have reported favorable clinical outcomes. Chen et al. (21) retrospectively analyzed 109 patients with stage IB2–IVA cervical carcinoma who were treated with IMRT, concurrent cisplatin-based chemotherapy, and high-dose rate BT (HDR-BT) for a median follow-up period of 32.5 months; they reported that 3-year OS, LFFS, and DFS rates were 78.2, 78.1, and 67.6%, respectively. In the RetroEMBRACE study that included 731 patients treated with definitive EBRT with or without concurrent chemotherapy, followed by IGBT (22), the 5-year OS and LC rates were 66.6 and 89%, respectively. A study retrospectively reviewed the experience of treating 250 patients with CC with EBRT and 2D low-dose-rate BT at The National Centre for Radiotherapy in Accra and indicated that 3-year OS and locoregional recurrence rates in these patients were 86 and 19%, respectively (10). In a recent update of the study conducted by Wang et al. (3), 1,443 patients with stage IB1-IVA CC were assigned to IMRT with a dose of 50.4 Gy in 28 fractions combined with ICBT and concurrent chemotherapy and had a median follow-up period of 32.2 months; the 3-year OS, DFS, and LC rates of these patients were 83.0, 75.0, and 87.4%, respectively. Notably, in many of these studies, patients were likely treated with either 4-field external beam radiation plans or 3D-CRT, which may have contributed to higher toxicity rates. Moreover, these studies did not conduct long-term follow-ups or report complications, thus requiring further observation and research. The results observed at our institution are similar to those published previously. Most of the cervical cancer patients in our study had stage IIB and IIIB. Our results showed that 3-year LC, OS, and DFS rates for patients treated with VMAT were 88.7, 80.5, and 65.4%, respectively. A previous study (21) found that the rate of locoregional failure was 13.8% and the rate of distant metastasis was 22.0% for patients with stage IB2–IVA CC. Our study results demonstrated that the rate of distant relapse was 18.7% and the rate of local failure was 10.6% for patients with stage IA–IVB CC in VMAT group.

Regarding radiation complications, the incidences of late toxicities observed in our study appeared to be lower than those reported in previous studies. In a trial conducted by Hasselle et al. (23), 4 and 5% of the cohort had grade 3 or 4 gastrointestinal and genitourinary toxicities, respectively. In particular, Chen et al. (24) reported that late grade 3 toxicity respectively occurred in the rectum and bladder of 2.4 and 3.6% of 83 patients with intact cervix treated with IMRT. Notably, in our study, we observed a significant benefit in terms of rectal and bladder complications in VMAT group, finding that only 0.9 and 2.1% of patients had chronic grade 3 or higher cystitis and enterocolitis, respectively. These findings indicate that VMAT could ensure dose distribution in target volumes while effectively reducing dose absorption in OARs. Acute grade 3 or higher hematological toxicity was experienced by 13.6% of the patients. Bone marrow cells are sensitive to radiation, related researches showed that radiation-induced bone marrow damage is dependent on both dose and volume (25, 26). Furthermore, a study conducted by Guo et al. (27) found that there was no statistical significance between VMAT and IMAT in radiation-induced myelosuppression. However, pelvic volume of low-dose irradiation and dose limiting should be considered to avoid the occurrence of severe acute hematological toxicity during the VMAT treatment plan. In the current study, pelvis has not been included in the routine OARs, thus more prospective controlled trials are needed to confirm the effect of VMAT on acute hematotoxicity. Our results indicate that the prescribed radiation dose of 45–50 Gy is reasonable based on the observation of relatively satisfactory clinical outcomes and tolerable treatment-related toxicities. In addition, we used freehand IC/IS BT that increased the dose delivered to the tumor target, thereby improving therapeutic outcomes.

This study has several limitations. The main limitation is the retrospective nature of this study with its inherent biases. Second, this was a single-institution experience and IMRT group had small sample size to demonstrate whether VMAT combined with IC/IS BT actually improved survival and reduced toxicity. Third, late toxicity was assessed based on a retrospective records, therefore, late toxicity rates may have been affected. Furthermore, it is difficult to draw firm conclusions that promising overall outcomes observed in our patients are attributable to VMAT because some patients (30.5%) in our study did not receive concurrent chemotherapy and we can not control all confounding factors. A large, randomized, multi-institutional study is needed to confirm the effectiveness of definitive VMRT for patients with cervical cancer.

To the best of our knowledge, this is the largest series of patients with CC who has ever been definitively treated with VMAT combined with IC/IS BT. We present our single-institution experience, 3-year LC, OS, and DFS rates were 88.7, 80.5, and 65.4% in VMAT group, respectively. In addition, patients treated with VMAT combined with IC/IS BT had lower incidences of chronic radiation enterocolitis.

## Data Availability

All datasets generated for this study are included in the manuscript and the Supplementary Files.

## Author Contributions

XC conceived of designed and supervised the study. YLin, YO, ZL, and KC collected the data and analyzed the data. ZL and YLiu provided technical assistance with the study. XC and YLin wrote the manuscript. All co-authors have reviewed and approved this version of the manuscript.

### Conflict of Interest Statement

The authors declare that the research was conducted in the absence of any commercial or financial relationships that could be construed as a potential conflict of interest.

## Supplementary Material

Supplemental Figure 1 Kaplan-Meier estimated of the overall survival (OS) **(A)**, disease-free survival (DFS) **(B)**, local control (LC) rates **(C)**, and distant metastasis free survival (DMFS) **(D)** of cervical cancer patients treated with definitive VMAT and IMRT.
