# Supplementary figures and images for: Clinical Outcomes of Volumetric Modulated Arc Therapy Following Intracavitary/Interstitial Brachytherapy in Cervical Cancer: A Single Institution Retrospective Experience
Source: Front Oncol. 2019 Aug 16;9:760. doi: 10.3389/fonc.2019.00760 (PMC6707003; doi:10.3389/fonc.2019.00760)

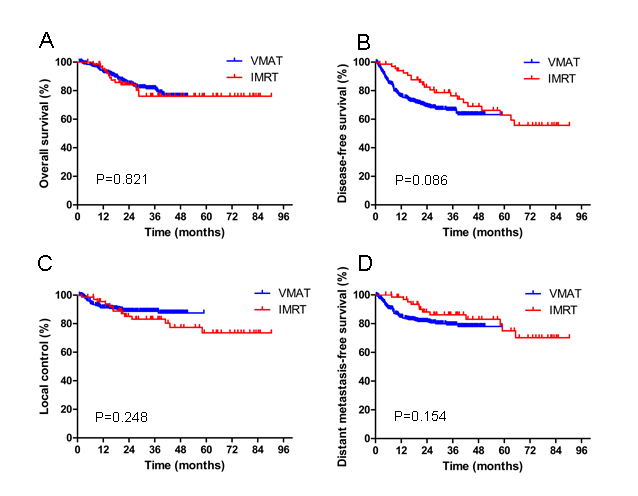

Supplement: Supplemental Figure 1 — Kaplan-Meier estimated of the overall survival (OS) (A), disease-free survival (DFS) (B), local control (LC) rates (C), and distant metastasis free survival (DMFS) (D) of cervical cancer patients treated with definitive VMAT and IMRT. [file Image_1.TIF]
